# Supplementary material for: SKGQA, a Peptide Derived from the ANA/BTG3 Protein, Cleaves Amyloid-β with Proteolytic Activity
Source: Biomolecules. 2024 May 15;14(5):586. doi: 10.3390/biom14050586 (PMC11118129; doi:10.3390/biom14050586)
Supplement: Supplementary file 1 [file biomolecules-14-00586-s001.zip › biomolecules-2815350-supplementary.pptx]

## Slide 1
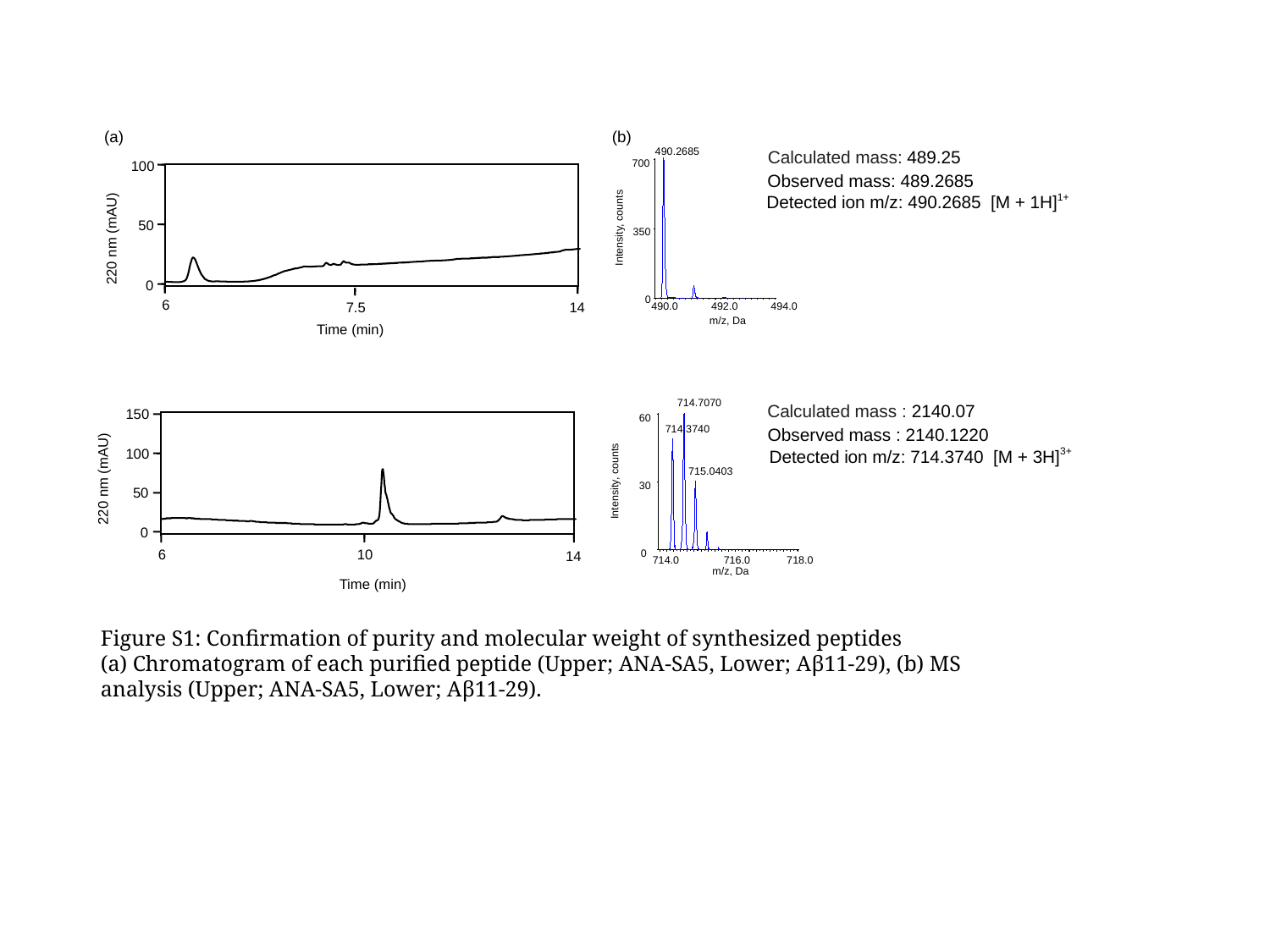

(a)
(b)
Calculated mass: 489.25
490.2685
700
Intensity, counts
350
0
490.0
492.0
494.0
m/z, Da
100
Observed mass: 489.2685
Detected ion m/z: 490.2685 [M + 1H]1+
50
220 nm (mAU)
0
6
7.5
14
Time (min)
Calculated mass : 2140.07
714.7070
60
714.3740
715.0403
Intensity, counts
30
0
714.0
716.0
718.0
m/z, Da
150
100
50
0
Observed mass : 2140.1220
Detected ion m/z: 714.3740 [M + 3H]3+
220 nm (mAU)
10
6
14
Time (min)
Figure S1: Confirmation of purity and molecular weight of synthesized peptides
(a) Chromatogram of each purified peptide (Upper; ANA-SA5, Lower; Aβ11-29), (b) MS analysis (Upper; ANA-SA5, Lower; Aβ11-29).

## Slide 2
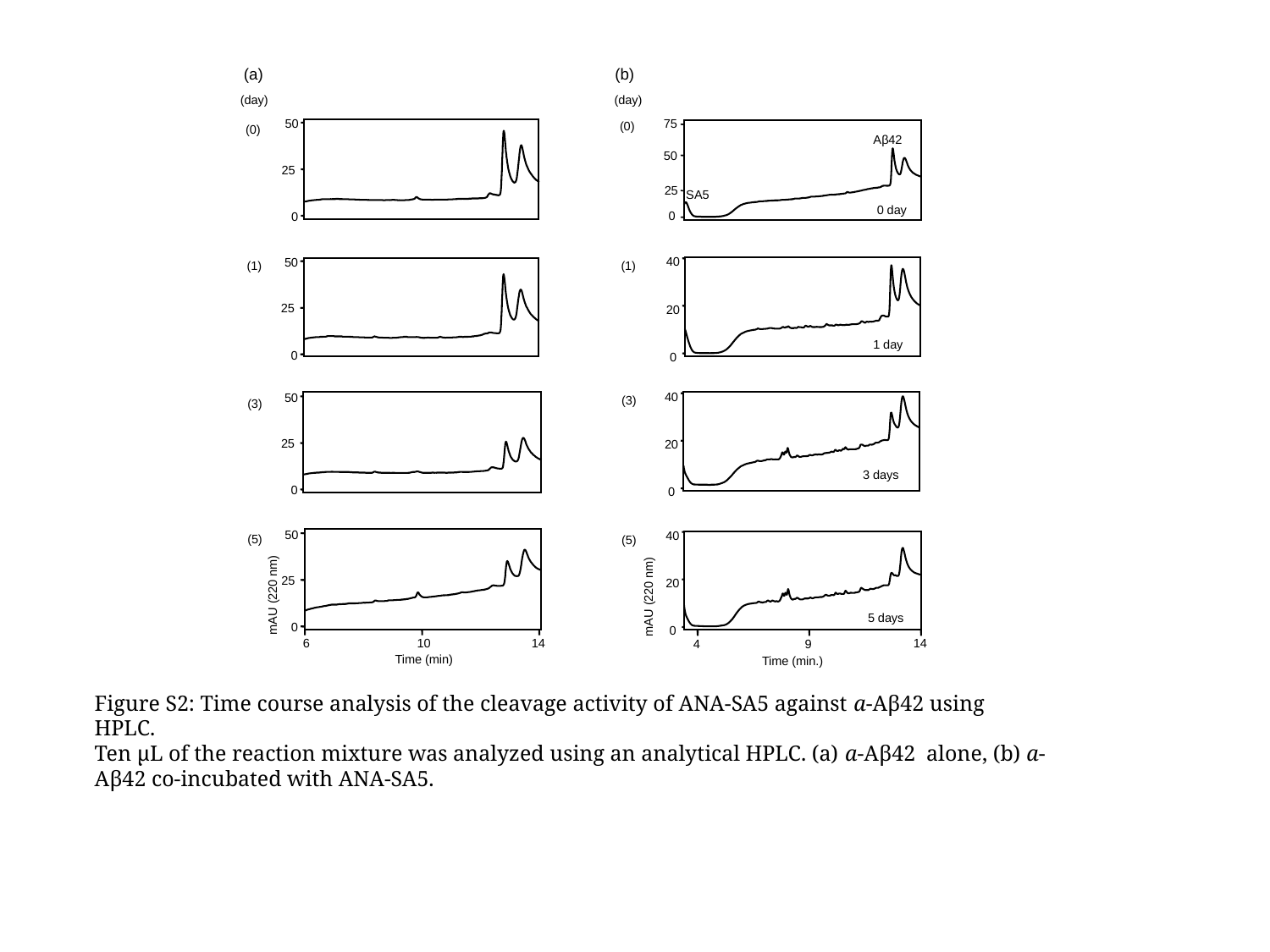

(a)
(b)
(day)
(day)
(0)
(0)
75
50
25
0
50
25
0
Aβ42
SA5
0 day
(1)
(1)
40
50
25
20
1 day
0
0
(3)
40
20
0
50
25
0
(3)
3 days
(5)
(5)
50
25
0
40
20
0
mAU (220 nm)
mAU (220 nm)
5 days
10
6
14
14
4
9
Time (min)
Time (min.)
Figure S2: Time course analysis of the cleavage activity of ANA-SA5 against a-Aβ42 using HPLC.
Ten μL of the reaction mixture was analyzed using an analytical HPLC. (a) a-Aβ42 alone, (b) a-Aβ42 co-incubated with ANA-SA5.

## Slide 3
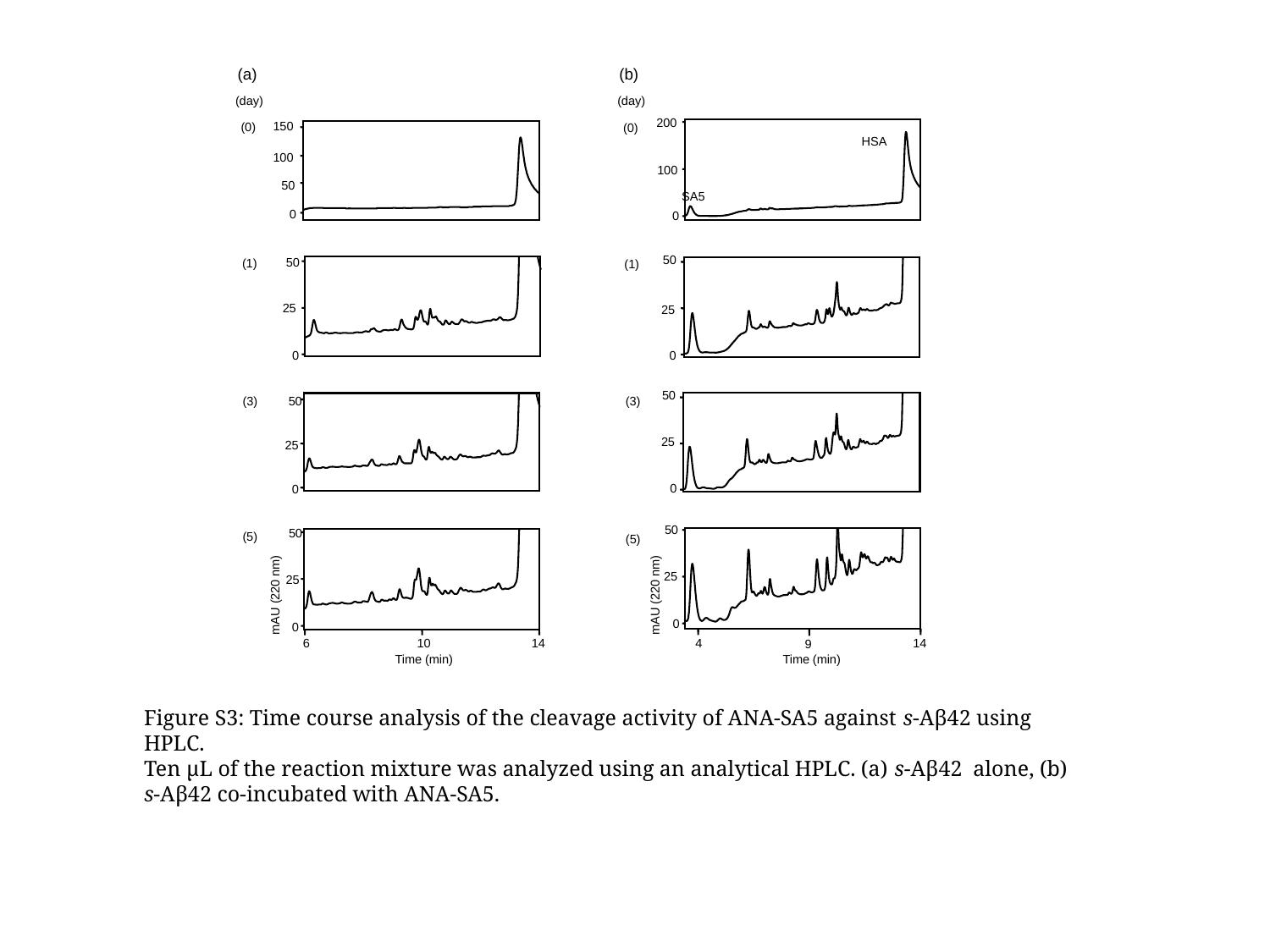

(a)
(b)
(day)
(day)
(0)
(0)
200
100
SA5
0
HSA
150
100
50
0
(1)
(1)
50
25
0
50
25
0
50
25
0
(3)
(3)
50
25
0
50
(5)
50
25
0
(5)
25
mAU (220 nm)
mAU (220 nm)
0
4
10
14
6
14
9
Time (min)
Time (min)
Figure S3: Time course analysis of the cleavage activity of ANA-SA5 against s-Aβ42 using HPLC.
Ten μL of the reaction mixture was analyzed using an analytical HPLC. (a) s-Aβ42 alone, (b) s-Aβ42 co-incubated with ANA-SA5.
